# Developing and Gathering Validity Evidence for an Instrument to Measure How High School Students Identify as Researchers

**DOI:** 10.1007/s11165-024-10194-1

**Published:** 2024-08-28

**Authors:** Linda Morell, Shruti Bathia, Bon W. Koo, Mark Wilson, Perman Gochyyev, Rebecca Smith

**Affiliations:** 1 University of California, Berkeley, (Berkeley School of Education), Berkeley, CA, USA; 2 University of California, Berkeley, (Lawrence Hall of Science), Berkeley, CA, USA; 3 University of California, San Francisco, (Science and Health Education Partnership), San Francisco, CA, USA

**Keywords:** Researcher identity, Authentic research experiences, Survey, STEM identity, Identity, Rasch analysis, Science identity

## Abstract

The authentic research experience, which provides students with meaningful collaborative research opportunities designed to promote discovery and innovation under the guidance of mentors, is increasing as a way to attract and engage students in STEM fields. However, despite the increase in authentic research experiences offered to students, there has been little research, particularly at the high school level, investigating students’ attitudes about themselves as researchers. To address this need, we developed a theory (or construct) for how high school age students self-identify as researchers and a companion survey to measure their identity. After three iterative development cycles, 823 high school students from diverse backgrounds were administered the 12-item survey, the Researcher Identity Survey—Form G (RISG). The partial credit Rasch model (1960/1980) was used to analyze the survey data. The results indicate that the survey identifies and locates high school age students as researchers validly and reliably along an easy to use and interpret scale. The survey holds promise as an important element for use in programs designed to broaden the entryway for students into the STEM disciplines.

## Introduction

Attracting more students into the science, technology, engineering, and mathematics (STEM) disciplines is critical to meeting future workforce demands and will require new approaches and new strategies. The authentic research experience, which is designed to engage students in iterative research practices guided by a mentor to generate new information (Deemer et al., 2021), is one such approach and this kind of experience has received support from federal agencies within the United States government as evidenced by the 2013 and 2018 reports by The Committee on STEM Education of the National Science and Technology Council. This focus at the federal level has brought added attention to efforts already underway by educators who seek to develop interventions that attract and retain youth so that they may ultimately embark on a STEM career. While there are many factors that contribute to students’ interest and persistence in STEM careers, research indicates that identity can be a critical factor in making a career choice. For example, Stets et al. (2017) found that “the identity process is the primary mechanism through which minority students choose a science occupation” (page 12). This highlights the importance of attending to the affective (non-cognitive) aspects of student development and specifically to identity.

While not all STEM careers involve conducting research-related activities, many do. Moreover, Marvasi et al. (2019) assert, based in part on previous research (Castello et al., 2015; Sweitzer, 2009), that “it is widely accepted that the development of a ‘researcher identity’ is critical to becoming a practicing scientist whether in a structured setting, such as an academic or private laboratory, or as a citizen-scientist” (p. 1).

Even though identity has been shown to be a primary driver of career choice and there has been increased interest and funding for authentic research experiences for high school students, little is known about “researcher identity.” Therefore, providers of research experience programs would benefit from a valid and reliable instrument to measure researcher identity. This research systematically investigates the theory (construct) of researcher identity and describes the development and validation of a measure of researcher identity. The study was guided by this research question: *How (and to what degree) can the construct of researcher identity of high school students be measured?*

## Literature Review

### Identity Formation

Social scientists consider identity to be a set of meanings that define who a person is in terms of their roles (role identity), group or category memberships (social identities), or as unique individuals (person identities) (Burke & Stets, 2009). Gee (2000) suggests that the shaping of someone’s identity includes the individual themselves, as well as how others see them. During adolescence and early adulthood, identities are being developed, shaped, and refined. These multiple role identities are structured and prioritized. A role identity that ranks higher in an individual’s identity hierarchy has a greater probability to be enacted (Serpe & Stryker, 1987). An individual’s identity is generally not confined to a single role. It is neither stable across time nor within a given timeframe. A person can want to be a part of many different communities all at the same time. In adolescence, students move between social contexts, and in each of these contexts the different parts of their identities shift and become more or less important. Although the process of identity development is an individual one, it is a process that is socially situated, so that individuals see themselves within and a part of the social world. Students’ actions then become an expression of their social identities because it is their social identities that organize their activity (Lloyd & Duveen, 1992). Therefore, opportunities to participate within and outside of school become critical and can influence the development and enactment of a student’s identity. Both school science and informal science programs such as authentic research experiences provide opportunities for students to explore nascent identities and assess how others react, which helps students test the fit of a nascent identity (Brickhouse et al., 2000).

### Science and STEM Identities

The science identity of students is rooted in the belief of their capability and can be aspirational in terms of what they want to do in the future (Brickhouse, 2001). According to Carlone and Johnson (2007), three dimensions contribute to science identity formation—*performance* of scientific practices, *recognition* both by the self and others, and the less visible *competence*—knowledge of science content. Experiences, whether in or out of school settings, especially those that provide opportunities for performance, recognition, and development of competence, can play an important role in strengthening students’ science identity. Youth who perceive science as significant to their lives, their future career prospects, and to society as a whole have higher science identities than youth who do not (Archer et al., 2012; Bennet & Hogarth, 2009).

However, in a large survey of sixth, seventh, and eighth-grade students, Hill et al. (2018) found that some students, particularly adolescent girls and students from backgrounds underrepresented in the sciences, had lower interest in science than others when the term “science” was used but found the same rate of what they termed “discovery orientation” among all students, regardless of gender or race and ethnicity. Questions probing discovery orientation asked students to indicate: (1) how much they like learning about new discoveries, (2) how curious they are about the world, and (3) how much they enjoy exploring nature. Those are all behaviors frequently associated with science or science interest. Studies like Hill et al. (2018) raise the possibility that using alternative wording for science, that are less encumbered by historical and cultural narratives that limit who can participate, may help a wider range of students recognize their inclination for, interest in, and ultimately identify with a variety of roles and careers in science. Research, and its derivative researcher, is one such alternative term.

Verdin and colleagues (Verdin et al., 2018) studied the STEM identities of high school students. Their perspective was that “students’ identity plays a pivotal role in their decision to pursue careers in STEM” (p. 32). So, their research explored the ontological beliefs of high school students, which paid special attention to how students describe a math person, science person, physics person, or engineer. The study investigated how the students’ descriptions influenced their facility and willingness to take on the role identities. For the study, researchers conducted interviews of 17 high school students (freshman to seniors) enrolled in chemistry and/or physics at two schools. Verdin et al. (2018) used an ontological approach for each STEM identity (what constitutes a science person, physics, person, math person, or an engineer?) instead of investigating how these identities develop. Verdin et al. (2018) generated multiple ideas and concepts such as “Integrated [identity],” “Curiosity,” and “Exploratory” that are suitable for leveraging across affective domains.

### Importance of Assessing Researcher Identity

Researcher identity can be seen as both broader and narrower than science or STEM identity. It can be considered *broader* because the “research” can span across all disciplines and methodologies (including STEM) while at the same time be considered *narrower* because it does not account for the whole of any one discipline. Because of this unique position visa-vis STEM, research(er) identity is an important variable to assess.

Assessing the researcher identity of high school students is important for several reasons. Authentic research experiences will primarily occur in research-driven educational programs, so measuring researcher identity is more closely aligned to aims and goals of those kinds of programs than measuring another (e.g., science or STEM) identity. Additionally, although STEM identities have been studied extensively, research indicates that for some students using those terms (i.e., “STEM” or “science”) may evoke less interest and therefore may limit participation. Using a less limited term, such as “researcher,” would avoid that possibility. For example, research (Tan et al., 2008, 2013; Calabrese Barton et al., 2013) with under represented populations, especially girls, has indicated that they may not identify with STEM even if they show promise of success in the field. In addition, studying the nature and expression of researcher identity is important in many of the same ways that studying identity within STEM contexts is important. For example, research shows that assessing STEM identity helps uncover motivating factors and mechanisms underlying students’ choices related to STEM activities and how their career aspirations develop (Archer et al., 2012; Hazari, et al., 2010; Shaby & Vedder-Weiss, 2020). Other STEM identity research shows that understanding how a student’s identity develops also helps the field understand how they understand their STEM-related experiences (Drayton, 2020). We predict that similar findings regarding the broader researcher identity may well hold, and the establishment of a valid and reliable instrument to measure it would be an important step to gathering such evidence. Finally, research conducted specifically about researcher identity exists for higher education students but not for high school students. For example, Costello et al. (2015) provides a sociocultural framework to conceptualize researcher identity for graduate students. The framework is described as a process of the self that accounts for both continuity of stable personhood over time and a sense of ongoing change.

We investigated several instruments before ultimately deciding to develop a new one. For example, we considered using instruments like the Science Motivation Questionnaire II (SMQ II) by Glynn, et al. (2011), the Science Attitudes Inventory by Moore and Foy (1997), and the STEM Career Interest Survey (STEM-CIS) by Kier et al. (2014). However, these instruments were deemed inappropriate for one or more of the following reasons:

- The focus was on a similar but different construct (e.g., motivation, attitudes toward science, or career interest in STEM);
- The targeted age group for the instrument was younger or older than high school;
- A Likert-based survey was developed, which can have various problems (Wilson et al., 2023; Carifio & Perla, 2008) that we wished to avoid;
- Previous studies relied on factor analysis instead of Item Response Theory models (IRT). IRT models acknowledge the categorical/ordinal nature of the item responses and allows for the modeling of step parameters.

Thus, we chose to develop a survey measuring researcher identity instead of science identity or STEM identity because the central aim of authentic research experiences is to provide students with ***research*** experiences within the context of a variety of STEM disciplines and leveraged some of the ideas and terminology from previous studies for inspiration as we conducted our study into researcher identity. Therefore, our goal was to define and develop a construct and accompanying instrument to determine how high school students (grades 9–12) self-identify as researchers since it is at this age that students begin engaging in authentic research experiences and begin to wonder about themselves by asking questions such as:

- Does the role of researcher fit me?
- Do I feel a sense of belonging in the research world?
- Do I perceive myself as a researcher?
- Do I see doing research in my plans for the future?

### Conceptual Framework

**Fig. 1:**
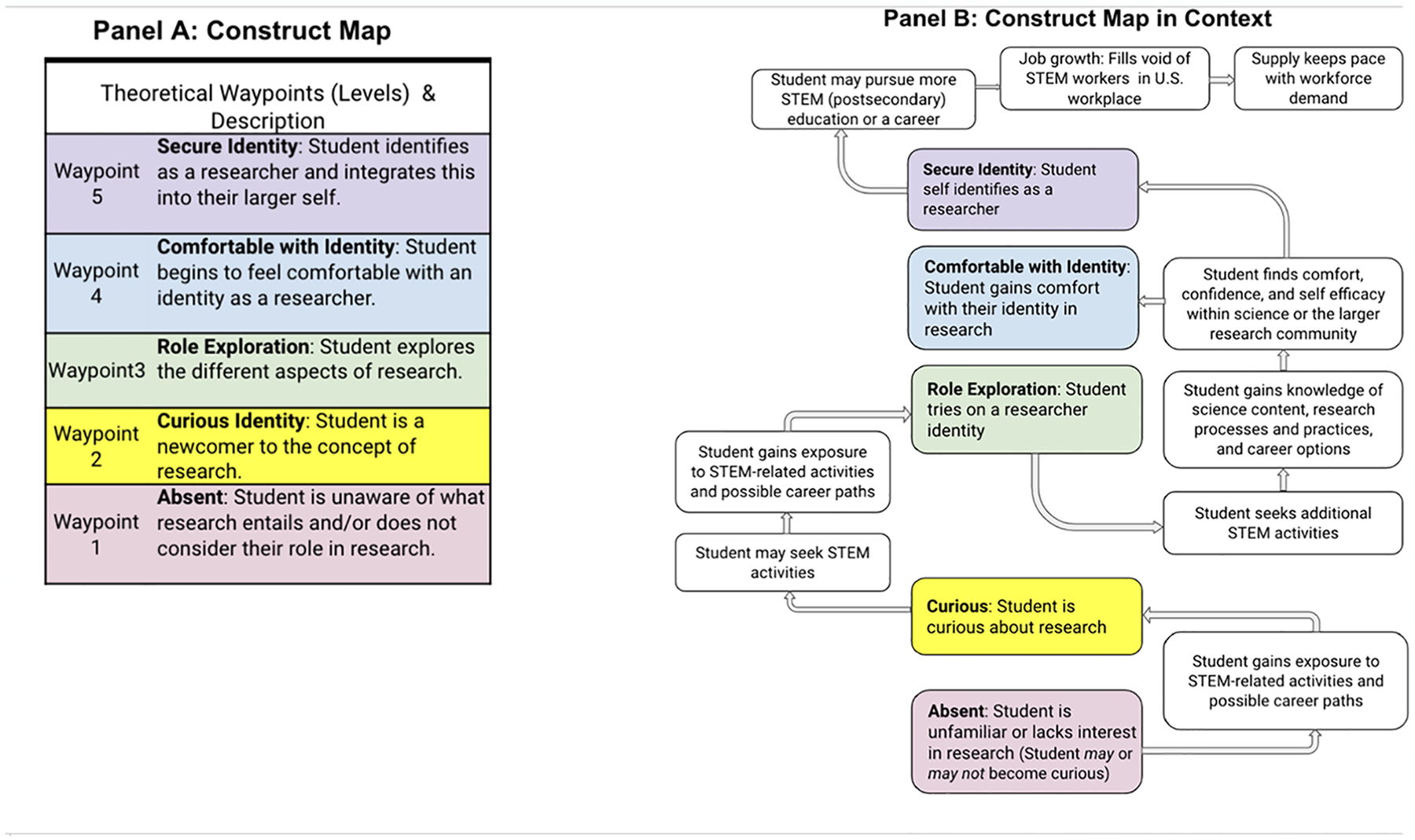
Theory (Construct Map) for Researcher Identity (Panel A) & the Construct Map embedded within context (Panel B) *Note*. Panel A shows the waypoints of the researcher identity theory, which make up the construct map for researcher identity. Panel B illustrates how the construct map is situated within the context of activities and actions that contribute to students’ growth.

We propose that researcher identity can be thought of as progressing over time through theoretical points along the way (waypoints) from “absent” to “secure.” The continuum should be viewed as a possible path of researcher identity development over time. The researcher identity construct shown in Fig. 1, emphasizes the developmental structure of the “researcher identity” progression. Panel A shown on the left side of Fig. 1 provides the theorized developmental waypoints that a high school student could move through as their identity as a researcher increases. Panel B shown on the right side of Fig. 1 shows the developmental structure within the high school context where a student is provided with and/or may seek out STEM-related research opportunities on their route to a possible STEM-related career.

### Points along the Way (The waypoints or levels of the developmental continuum)

The waypoints were created based on prior theories of identities in science (Brickhouse et al., 2000; Costello et al., 2015), STEM (Kim et al., 2018), gender (Lloyd & Duveen, 1992), and in general (Gee, 2000).

At the earliest waypoint of the continuum, we propose that there is the “Absent” waypoint. Students at this waypoint generally do not consider themselves or their role in research; they may or may not progress along the trajectory. Next in the theory progression is the “Curious Identity,” which suggests that the student is a newcomer to the concept of research and is curious to learn more. This waypoint was based on the research of Costello et al. (2015) and Brickhouse et al. (2000). Next, is “Role Exploration,” which suggests the student is exploring the different aspects of research. After Role Exploration comes “Comfortable with Identity” in which the student shows signs of beginning to feel comfortable with their identity in research. This waypoint was informed by a report (Auchincloss et al., 2014) about assessment of Course-Based Undergraduate Research Experiences. Finally, there is the “Secure Identity” or “Integration of Identity.” This is the highest waypoint theorized on the construct map and is described as the student identifies as a researcher and integrates this into their larger self. This waypoint was informed by Lloyd and Duveen’s (1992) research. A student at the Secure Identity location is theorized to have a strong voice and connection to the research community. In addition, the student would find joy and excitement in research and aspire to a career in research.

### Strands of the Construct

To address the whole of the researcher identity theory, we again needed to borrow from other domains, like STEM identity and science identity, to varying degrees as well as the scant researcher identity literature focusing on graduate students like the work of Madikizela-Madiya et al. (2013). The concept of researcher identity is hypothesized to be a unified idea made up of four strands: self, community, agency, and fit & aspiration.

The “Self” strand relates to the students’ current self-conceptions of themselves as researchers. Adolescence is a time when identity formation is flexible and multifaceted. Oftentimes, there can be a “mismatch between popular representations of science, the manner in which it is taught, and the aspirations, ideals, and the developing identities of young adolescents” (Archer et al., 2010, p. 618). We view this perspective as an important consideration in addressing the self strand of researcher identity and see the student’s current view of the self to be important for program providers to understand.

The “Community” strand of the construct indicates a sense of belonging to a community of researchers. Notable researchers (Brickhouse & Potter, 2001, and Carlone & Johnson, 2007) have investigated the importance of community and how becoming a part of a particular community (often a science learning community) is informed and reflective of one’s own knowledge and practice within that community. In this study, we are interested in understanding how a high school student feels within a community of researchers. This would encompass both a peer research community and belonging to a larger professional community.

“Agency” is defined as the degree to which a student feels empowered to enact change through research. The agency aspect of the construct is informed by prior research on persistence (Graham et al., 2013) and self-efficacy and self-concept of multicultural students (Best et al., 2019). Self-efficacy, according to Bandura (1997), is the belief of the individual that they can plan and implement a course of action successfully while self-concept (Best et al., 2019) “is represented as judgments of global self-constructed belief that reflects one’s view of accomplishments, capabilities, values, body, others’ responses, events, occasions, and possessions” (p. 102). Both, but especially self-efficacy, are strong performance domain factors in education. Of particular interest is the idea of “researcher voice.” We define “researcher voice” as the extent to which the student feels empowered to speak about their research. This aspect of agency is important because a student’s voice is a way for the student to recognize and have others recognize them as they enact an identity role (Carlone & Johnson, 2007).

The “Fit & Aspiration” strand includes ideas about the “future self,” and indicates a student’s interest in research as a career path and is seen as important since high school age students are thinking or beginning to think about their future. Research suggests that students, in particular girls from nondominant backgrounds in the sciences and engineering, view their possible future selves within an argument framework that is ongoing, cumulative, and contentious which may shift their future goals and career choices (Barton et al., 2013). Archer and colleagues (2013) conducted a longitudinal study of ten to 14 year old females to explore their aspirations and possible future career choices. The researchers (Archer et al., 2013) argue that females holding perceptions of people in science as brainy, not nurturing, and geeky prefer to not identify with them and do not aspire to careers in science. Boucher et al. (2013) provide a comprehensive review of the impact of these stereotypes on STEM interest and career aspirations in girls and minoritized students. We saw this as an important element to capture through the instrument since (1) the STEM fields lack diversity and (2) because adolescents’ identities are malleable.

### Context of the Study

For high school students the most relevant community is that of the school itself (Brown et al., 1989). However, the activities students are offered at school often have little connection to communities outside of school, and generally, students are not engaging in actual science, instead they are engaging in “school science.” To address this shortcoming in school science, the Science & Health Education Partnership (SEP) at the University of California, San Francisco developed the San Francisco Health Investigators program (Koo et al., 2021). The program is funded by the National Institutes of Health (NIH) and is designed to engage students in real-world research-related activities with a focus on health. During the year-long program, 20 students are introduced to ethical and methodologically-sound research procedures and engage in authentic practices of science and engineering, as defined by the Next Generation Science Standards (NGSS Lead States, 2013). Students engage in a competitive process to enter the year-long program and a new cohort is chosen each year. Upon acceptance, each year a cohort of 20 high school students from traditionally marginalized populations in STEM participate in a month-long summer intensive (9am–3 pm daily) and then monthly follow-up meetings during the following academic year. Core to the San Francisco Health Investigators program is a community-based participatory research (CBPR) model (Israel et al., 2001). Students work as a community throughout their entire research experience as they conduct original research in their home communities and use their findings to inform the development and dissemination of health messages. The program provides opportunities for students to perform research tasks; recognize what they, themselves, have accomplished and be recognized by people in the research community and family members; and to develop knowledge and skills of both the health topic and research process. These principles reflect the ideas set forth by Carlone and Johnson (2007) as being important for identity formation.

## Method

We used the *Standards for Educational and Psychological Testing* (*Standards*, AERA et al., 2014) to frame our validity argument and used the construct modeling approach (Wilson, 2023) to guide our investigation. Using the construct modeling approach, we collected reliability evidence and validity evidence based on test content, response processes, internal structure, and relations to other variables; and evidence of fairness (e.g., differential item functioning or DIF), just as they are listed (as “sources”) in the *Standards*. (Note that we did not collect evidence about consequences, as there was no actual usage and hence no consequences at the point where we had just finished collecting the validity evidence.) The method section is organized and presented by the building blocks as specified in the construct modeling approach.

### Instrument Development

**Fig. 2:**
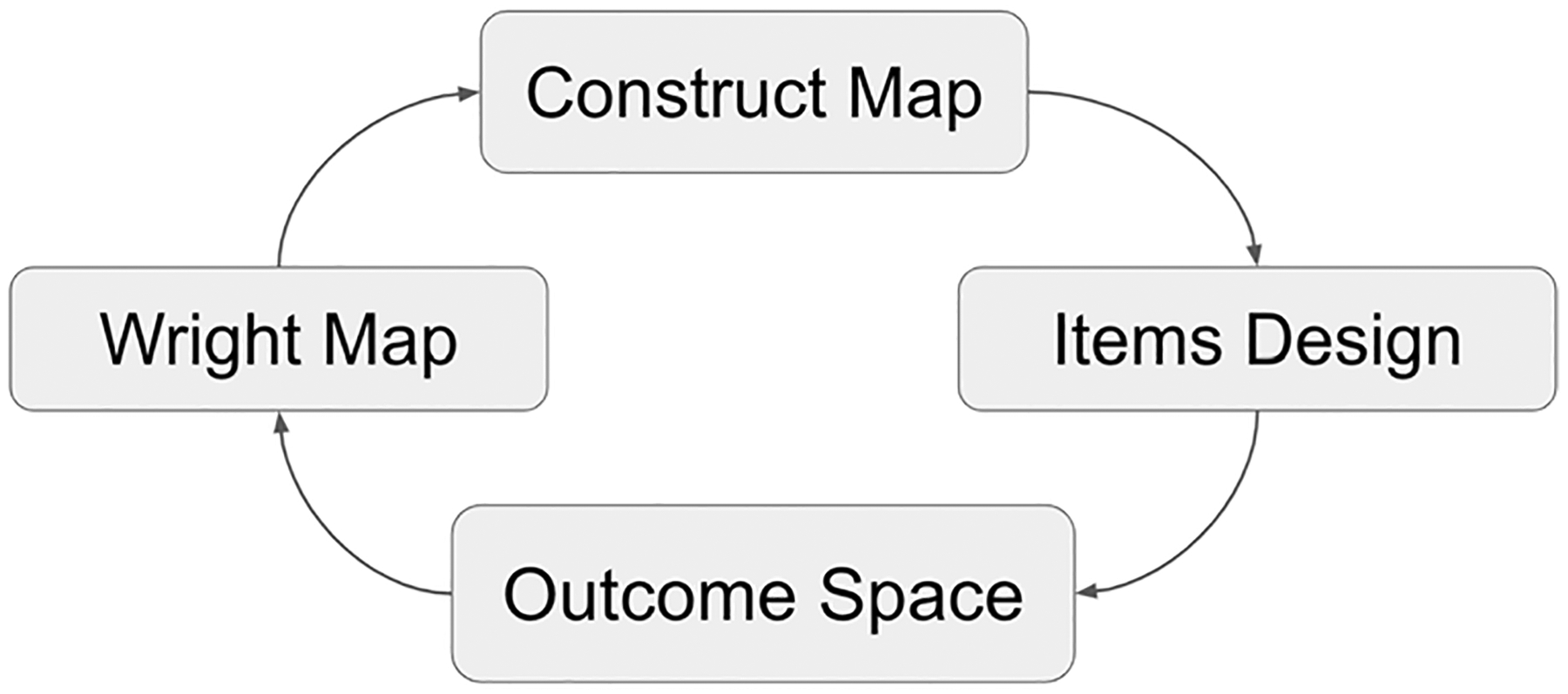
The Building Blocks of the Construct Modeling Approach

The construct modeling approach integrates four building blocks into a comprehensive instrument-development system, and the methods are derived from a well-established system for developing assessments. The four building blocks are named (1) the construct map, (2) item design, (3) outcome space, and (4) the Wright Map (Wilson, 2023). As shown on Fig. 2, these building blocks represent steps in a cycle of development, which can be repeated several times in order to arrive at a sound measurement tool.

**Fig. 3:**
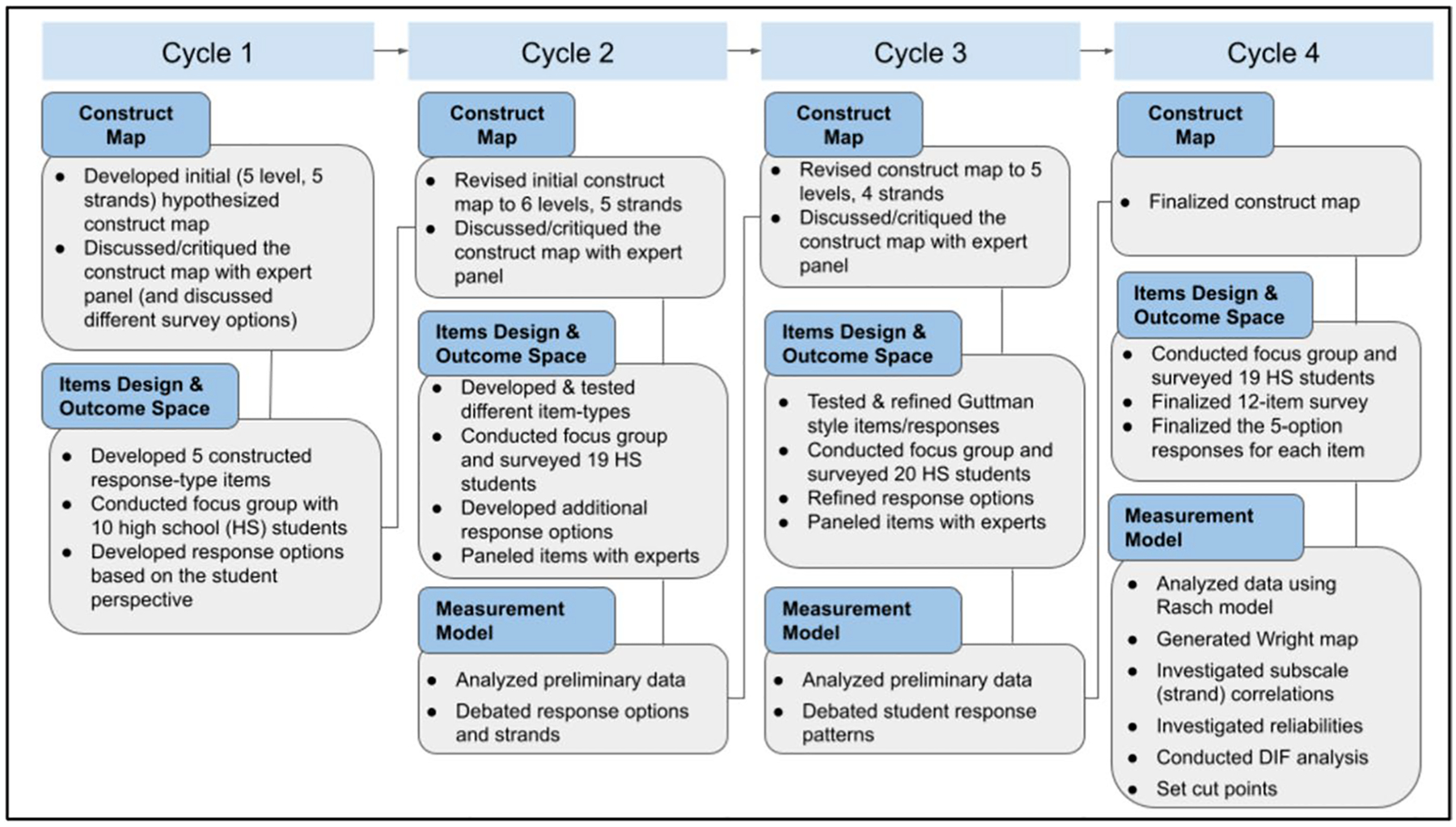
Flow Chart of the Development Procedure

We engaged in an iterative process of creating a construct, developing items using data from focus groups and surveys, and testing items through computer-based administrations; analyzing the data from the administrations; modifying and reorganizing response options; and modifying the construct. Each step of our research followed the construct modeling approach (Fig. 2) and is briefly described in Fig. 3. For this study, four iterations of the approach were necessary.

### Developing the Construct Map

**Fig. 4:**
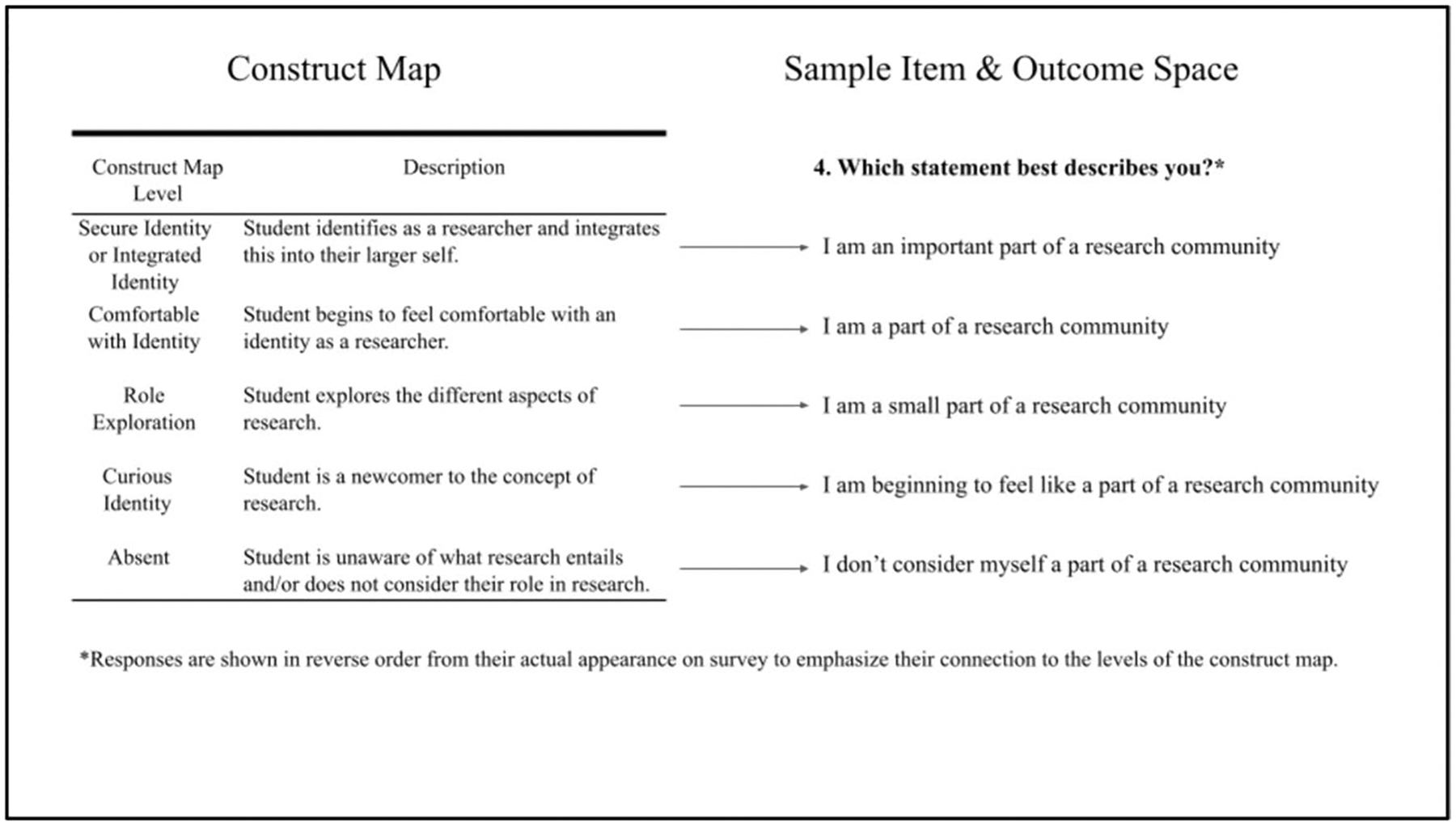
Researcher Identity: Construct with Sample Item & Outcome Space

A construct map is a unidimensional (one-dimensional) latent variable shown as a hierarchical complexity of skills (or attributes) that respondents are expected to progress through for the target construct (Chi et al., 2022). It is the explanation of the theory or construct and forms the basis of the validity argument for the internal structure of the test (or in this case the survey). It provides an ordering of qualitatively different *waypoints of the feature of interest* (i.e., self-identification in research) focusing on one characteristic derived in part from research into the underlying structure of the domain, and in part from professional judgments about what constitutes higher and lower waypoints of performance or competence. Construct maps are informed by empirical research about how individuals perform in practice (NRC, 2001). The construct map for the Researcher Identity theory is shown on the left side of Fig. 4.

The Researcher Identity construct was developed through an iterative design process that involved the close collaboration of the research and program teams involved in the project. The teams collaborated to develop a theory for how high school students develop a sense of researcher identity and discussed the theory with a diverse group of scientists and health professionals. The initial work was based on a review of identity literature and the needs and goals of the program. The construct and items were developed, reviewed, refined, and grouped into qualitative themes (strands—self, community, agency, and fit & aspiration) that identified the areas for possible growth in a student’s researcher identity. Focus groups and survey results from students led to further refinements of both the items and the composition of the strands. In addition, data collected through these methods helped to align the theoretical waypoints (e.g., absent, curious identity, role exploration, and comfortable with identity as shown on the left side of Fig. 1 and Fig. 4) to each item’s response options.

### Creating Items and Response Options (Outcome Space)

The second building block, called the items design, includes the questions, performances, or other indicators asked of a respondent to provide empirical evidence related to the waypoints of the construct map. The third building block, called the outcome space, defines a way of valuing and interpreting responses and assigning them to a waypoint on the construct map.

The right side of Fig. 4 shows a sample item (Item 4) and arrows indicate how the outcome space maps onto waypoints of the construct map. Response categories for each item target one waypoint of the construct map. Because the items were designed to reflect Guttman’s response format (Guttman, 1944), the outcome space is ordered to indicate the cumulative nature of the options design. This variety of item structure was chosen over other structures commonly used in surveys [e.g., the Likert (1932) format of strongly disagree to strongly agree] to take advantage of the affordances of the format to allow researchers to gather and interpret evidence based on internal structure (construct) validity easily. This choice of item type was supported by an article (Wilson et al., 2023) comparing Likert and Guttman response formats.

After iterating through the first three building blocks of the construct modeling approach, the final version of the RISG contained 12 items. Each item consisted of a stem and five cascading response options from least to greatest identification as a researcher. The full survey with a description of the instrument and scoring guide can be found in the Supplemental Materials.

### Connecting the Elements (Wright Map)

The construct map, items, response options, and respondents were calibrated using the fourth building block as specified by Wilson’s (2023) model for validation. Conquest 5.0 (Adams et al., 2020) was used to complete the analysis. We provide our findings on the qualities of the construct and the survey in the next section. Attention was paid to the technical aspects of the instrument to ensure valid, reliable, and fair use and interpretation of the survey results. We chose to analyze the data using the partial credit model (PCM) because it is a member of a family of measurement models that all share the possibility of “sample-free item calibration and test-free person measurement” (Wright & Masters, 1982, p. 38). The model provides a valuable approach to understanding survey validity by focusing on the fit of the individual item and step to the latent variable model (Wright & Masters, 1982). The model transforms raw information (item scores and person estimates) into logits which yields item scores and person ability estimates on the same interval scale.

We also investigated fairness using a differential item functioning (DIF) analysis for the gender and race variables. DIF was investigated using ConQuest 5.0 (Adams et al., 2020). Within the Rasch model, the test for DIF is specified by allowing the overall item difficulty to differ across demographic categories while controlling for the mean differences in the overall construct. An item is considered biased if, for example, two respondents from different groups have different probabilities of answering in the same response category even though they have the same amount of the latent trait.

### Samples

#### Students—Focus groups and formative surveys

Data were collected from a different group of students each year for four years. During the first year, 10 students from a diverse local high school in the United States comprised the first focus group. For the first round of data collection, students asked what “researcher identity” meant and how they might identify as a researcher. Next, students were asked to complete a nascent version of the survey. After the first year, the San Francisco Health Investigators program was fully operational so the approximately 20 students per year who participated in the program provided focus group data. To collect these data, students were asked to complete a draft Researcher Identity survey, then engage in a semi-structured focus group about the contents of the survey. This process was repeated three times. Procedures were based on standard methods (Krueger & Casey, 2009). In our case, four researchers conducted the focus groups. One researcher took the role of the moderator while another researcher took notes and ran the audio-recorder. Two additional researchers were available to distribute and collect materials, make observations of the group, and provide clarifications as necessary. Each focus group started with an opening question answered round robin style by each participant and was followed by an introductory question. Both of these early questions were carefully designed to provide a welcoming and open space and to warm up the participants so they felt comfortable talking and sharing their thoughts. Next, we asked a set of transition questions to allow participants to envision the topic of researcher identity through a broad lens. Those questions were followed by the key questions which delved more deeply into the participants’ own researcher identity, the bounds of identity, and the contents of the survey. Finally, we asked some ending questions to clarify actionable comments regarding the survey, enable participants to reflect on the session and their and their peers’ comments, and bring closure to the discussion.

A total of 58 students participated in the process over the four years. These students entered the San Francisco Health Investigators program through a competitive application process where teachers in U.S. state of California were asked to nominate students to apply to the program. Asking teachers to identify students who may not pursue a science research experience on their own gives students who have faced obstacles in the past an opportunity to move beyond those challenges and ensures the program with a diversified cohort yearly. At least 60% of program participants across the cohorts came from families where neither parent had a college degree. Many of the students are themselves immigrants or have parents who are immigrants. Most meet the federal definitions of low-income. At least two students experienced homelessness in their lifetimes and others in the program had incarcerated family members and/or are from families impacted by gun violence. Participants ranged in age from 16 to 18 years old.

#### Students—Calibration Sample

A diverse group of 823 high school students from the west coast of the United States provided responses to all items for the analysis. An additional 36 students were excluded from the analysis because of incomplete data. Students were recruited through a listserv of high school teachers, so the sample was one of convenience. It was necessary to collect empirical evidence from students with a wide range of researcher identities in order to identify and distinguish between the theoretical waypoints of the construct. Table 1 shows the demographic characteristics of the sample. Note that in some instances students completed the survey but did not provide full demographic information.

#### Panels of Experts

Expert panels were convened three times to provide feedback and suggestions on the larger program and the instruments under development, which included the Guttman-styled Researcher Identity Survey (RISG). Expert panelists also helped evaluate the suitability of existing surveys, interpret quantitative results, and set cut scores. Panels included experts in informal and formal science education, in psychometrics, and in science identity. In addition, panelists were experts in particular fields of science and most were members of minoritized groups. To evaluate the suitability of existing surveys, six experts were polled. To interpret the quantitative results and set cut scores six experts engaged in the “standard setting based on item response maps (Wright Maps)” procedures based on the text by Wilson and Draney (2002).

In summary, we use the construct modeling approach to investigate the validity of the Researcher Identity Survey Form G (RISG). Through this approach we collected the types of validity evidence identified in the *Standards* (AERA et al., 2014). Specifically, we collected data from experts during panel sessions to investigate the test content (and to inform partially our internal structures investigation) of the instrument. We collected data from high school students during focus groups as evidence for response processes. We collected survey data to investigate the internal structures of the RISG; we collected survey data from high school students using the SQM-II (Glynn et al., 2011) in order to collect evidence based on relations to other variables. We also used the survey data collected via the RISG to investigate the reliability and fairness of the instrument.

#### Findings

Reliability evidence and validity evidence (based on test content, response processes, internal structure, and relation to other variables and fairness) as outlined in the *Standards* (AERA et al., 2014) were investigated using the construct modeling approach (Wilson, 2023).

#### Reliability

The reliability of the person estimates within each of the stands ranged from 0.85 to 0.87 as shown on Table 2. The Self and Agency strands and the Community and Agency strands are the most highly correlated at 0.98 (each pair), but all subscale correlations are quite high as anticipated because we theorized them to be partial aspects of one construct—the researcher identity construct. In addition, the estimated reliability for the Researcher Identity (i.e., all items together) is 0.91 (coefficient alpha). The *expected* a posteriori (EAP) reliability is also 0.91. The reliability information provides evidence that people are answering items within the subscales in a consistent way.

#### Validity evidence based on test content

Before deciding to develop the instrument, researchers investigated other possible instruments like the Science Motivation Questionnaire II by Glynn et al. (2011); the SAI by Moore and Foy (1997); and the STEM Career Interest Survey by Kier et al. (2014). We used Lynn’s approach to determine content validity (1986) and Wilson and Draney’s (2002) approach to investigate the representativeness and relevance of the survey. During the first panel session, the existing instruments were discussed and deemed unsuitable for several reasons including, and most importantly, that they were not specifically designed to measure the researcher identity of high school students. Panelists agreed on the need to develop a new instrument during this first panel meeting. During the second meeting, researchers presented the construct and items (including the response options) for panelists feedback. A summary of changes and a revised instrument was provided to panelists for approval before final use. At the third meeting, six panelists judged that the items were found to tap the researcher identity construct, the response options of the items aligned with the waypoints defined on the construct map, and the content validity of the final survey’s content aligned with the researcher identity construct.

#### Validity evidence based on response processes

From focus group data, it was found that students had varying definitions of the term “researcher.” Therefore, researchers developed and tested a definition for the term “researcher.” Based on literature about science and STEM identities, the following definition was crafted and iteratively refined as “*A ‘researcher’ is defined as someone who conducts an organized and systematic investigation on a topic or question related to a scientific field.”*

The wording for items and response options for the RISG were accurate and clear and the block design in which a question stem is followed by five progressively more difficult response options was understandable and represented the theoretical waypoints as intended based on the results of the final round of focus group data collection.

#### Validity evidence based on internal structure (Construct Validity)

Choosing the measurement model is important because the model is the mechanism in which the items and response options relate back to the construct map and thus complete all the necessary steps specified in the construct modeling approach. Multiple pieces of evidence are necessary to understand the internal structure of the instrument model fit being one. We compared the one-dimensional model to the multidimensional between-item model (see Table 3). The multidimensional model specified four dimensions. Each dimension was based on one of the strands (i.e., self, community, agency, and fit & aspiration) theorized to comprise the researcher identity construct.

The one-dimensional Rasch model is a special case of the multidimensional Rasch model. The difference in deviances obtained from the estimation of the two models is evaluated as a chi-square statistic, with the difference in the number of parameters as degrees of freedom (shown in Table 3). Note, however, that dimension-specific variances cannot be nonnegative, and hence the null hypothesis is on the boundary of the parameter space. To adjust for it, the conservative test is obtained by dividing the resulting p-value by two (Rabe-Hesketh & Skrondal, 2012). Based on the results shown on Table 3, the difference in deviances (i.e., the chi-square statistic) is 308 (24,285–23,977) with nine (58–49) degrees of freedom, which is statistically significant at 0.01 level. However, as can be seen on Table 2, the correlations are so close to 1.00 that according to Wilson (2023) we would not, for any real-world reason (i.e., effect size) distinguish between them. In terms of statistical significance, we found that the multidimensional model fits better than the one-dimensional model, but we do not see that that supersedes the effect size. Further, the decision on which model to choose was motivated by the practical relevance of the model differences. Very high correlations between the dimensions imply that the additional complexity of the multidimensional model may not yield sufficiently distinct insights to justify its use over the simpler one-dimensional model. The decision to choose the one-dimensional model, despite the better statistical fit of the multidimensional one, reflects the most prudent way to balance fit, interpretability, and utility. We concluded that the added complexity does not offer practical benefits to outweigh the simplicity and ease of interpretation provided by the one-dimensional model (Wilson & Gochyyev, 2024). Hence, the conclusion to present the one-dimensional model.

Table 4 shows the response model information for the items, including the item difficulty estimations based on the partial credit model (PCM, Wright & Masters, 1982), the standard errors of the estimates, the weighted mean squares (fit indicators), and the corresponding *t*-statistic for the twelve items.

Items with low mean square values indicate items with a lower than expected variability where scores of high mean square indicate greater variability than expected results. According to Adams and Khoo (1996), in terms of effect size, a weighted fit mean square between 0.75 and 1.33 is generally acceptable. Item 2 is the only one outside the range, and this item has a statistically significant *t*-statistic as well (i.e., *t* is greater than 1.96). Given the relevance of the item to the construct, the panel decided to continue to include this item, but monitor it in further rounds of data collection.

**Fig. 5:**
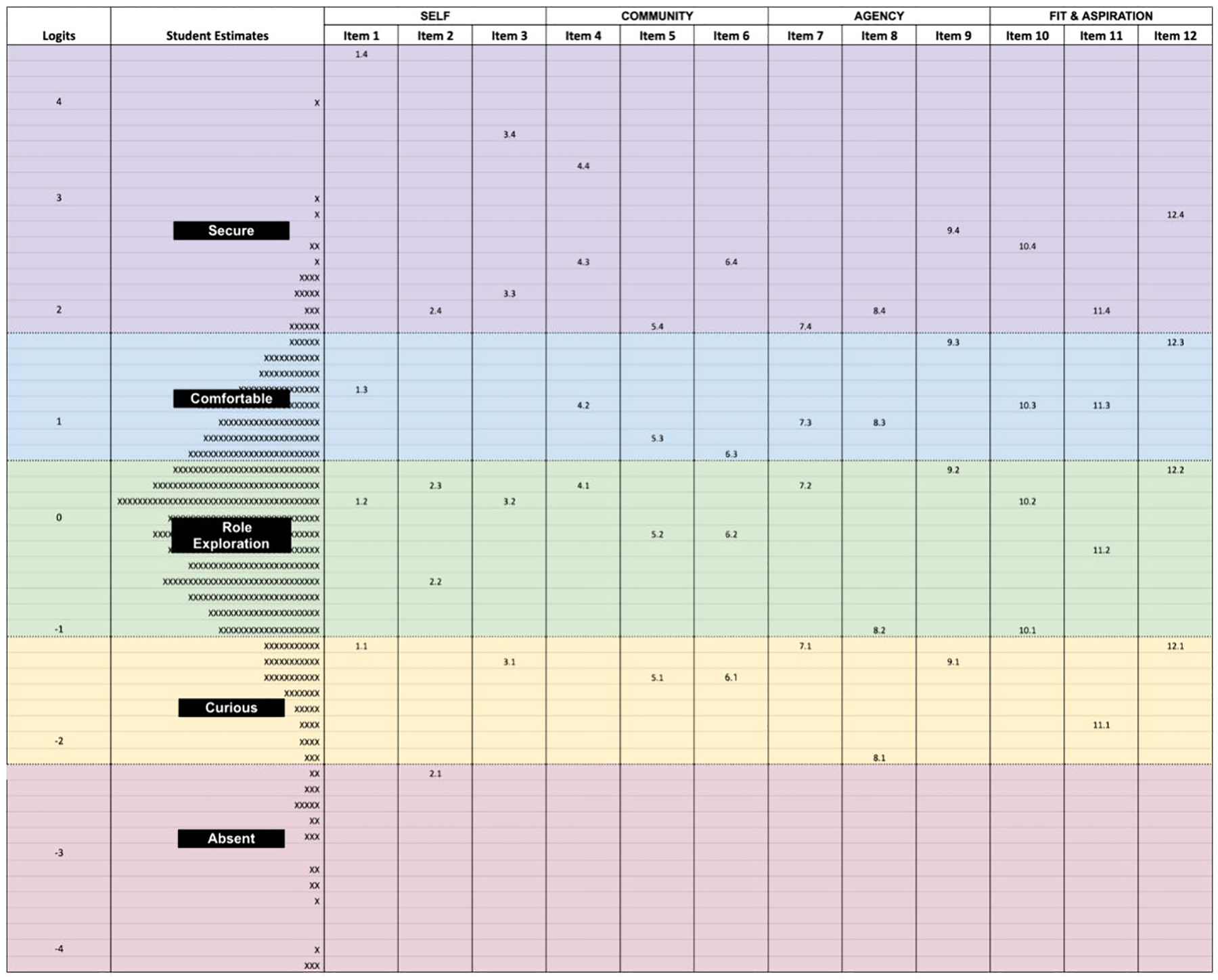
Wright Map: Person Estimates and the Items’ Thurstonian Threshold Location Each ‘X’ represents 1.5 cases

The Wright map (see Fig. 5) provides a graphical representation unique in measurement because the respondents’ (students’) ability estimates and the item difficulty locations are presented together on the same (logit) scale. The map provides insight into how the empirical evidence corresponds to the theory (shown on the left side of Fig. 4). The Thurstonian thresholds presented on the Wright map represent logit values at the 50 percent chance of answering at a given waypoint or below.

If the theory differentiating the waypoints functions as anticipated, we would expect that the item thresholds would be spaced fairly equally. The map shows that overall, the item spacing corresponds to the theoretical waypoints. As can be seen in Fig. 5 this is generally true with each threshold being spaced approximately 0.5 logits or greater apart from each other. For example, the steps for Item 1 are 1.1, 1.2, 1.3, and 1.4, with the first number representing the item number (1) and the second number representing the steps (_.1, _.2, _.3, and _.4). In addition, it would be anticipated that generally all of the first steps, second steps, third steps, and fourth steps would be found within a unique logit range, which is also generally true for the researcher identity Wright map.

The bands shown in Fig. 5 indicate the boundaries among the theoretical waypoints, which are shown on the left side of Fig. 4. Students at the bottom of the map appear to not self-identify as a researcher. Those student estimates fall in the logit range of −2.2 and below and are identified to be in the “Absent” range. A student with ability estimates within the “Absent” range is defined as “Student is unaware of what research entails and/or does not consider their role in research” according to the theory of development or construct map as shown on the left side of Fig. 4. The students with estimates falling in the logit range of −2.2 to −1.1 would be considered “Curious” (e.g., the student is a newcomer to the concept of research), while students with estimates in the logit range of −1.1 to 0.6 would fall in the “Role Exploration” category (e.g., the student explores the different aspects of research). Finally, students in the highest two categories are students in the “Comfortable with Identity” category (e.g., the student begins to feel comfortable with an identity as a researcher) with estimates in the logit range of 0.6 to 1.8, and the “Secure or Integration of Identity” category (e.g., the student identifies as a researcher and integrates this into their larger self) with estimates in the range above 1.8 logits.

To arrive at the banding, the panel members (during the third panel meeting) discussed the qualitative differences between each waypoint (e.g., absent, curious, role exploration, comfortable with identity, and secure or integration of identity) and then looked at the Wright map to find the different locations for each waypoint during the final panel. During that panel session, the group engaged in a back-and-forth standard setting session as specified by Wilson and Draney (2002) and ultimately arrived at distinctions or cut points between the waypoints. This was a fairly straightforward task because the Thurstonian thresholds were fairly evenly spaced showing the relationship between the response options and construct map. This was true except for Item 4, which appears very difficult on the Wright map. Item 4 asked students how much they considered themselves to be a part of a research community. Because the calibration sample was composed of high school students, in general, most were not a part of a research community. Therefore, this item appears more difficult, but when used with a sample of students participating in an authentic research experience this would likely not be the case. In the Rasch family of models (including the partial credit model used here), the sum score is a sufficient statistic. In other words, the order of respondents one would obtain from ordering based on the sum score is identical to the order one would obtain from ordering based on location estimates on the latent variable (assuming we do not have missingness). While raw scores are not interval scale, estimates on the latent variable are assumed to be on the interval scale. While we developed the waypoints on RIS-G using the logit scale (location estimates on the latent variable), the one-to-one mapping (between raw scores and these locations) allows us to map raw scores to these waypoints and thus simplifies the interpretation process for teachers and others.

This process and the resulting cut points provide strong evidence that the items (and instrument as a whole) can identify a student’s waypoint of identity in research.

#### Validity evidence based on relation to other variables

While an equivalent instrument for high school students does not exist, the Science Motivation Questionnaire II (SQM-II, Glynn et al., 2011) was chosen to test for convergent validity evidence. The SQM-II is a widely used instrument to identify the motivation of college students to learn science. The SQM-II is a 25-item survey with five possible response options: Never, Rarely, Sometimes, Usually, and Always. While not an exact match due to the targeting of college students, the motivation construct is similar in nature to the researcher identity construct. Thus, we would expect a positive correlation.

The analysis showed a positive correlation between student responses on the SMQ-II and the RISG when analyzed using the *expected* a posteriori, EAP, estimates (r = 0.79, n = 18, p < 0.05) and the summed scores (r = 0.73, *n* = 18, *p* < 0.05). We find convergent evidence between the two measures given that the constructs are of a similar nature and the correlations between student responses on the instruments were sufficiently strong.

#### Fairness

We conducted a differential item functioning (DIF) analysis to investigate bias for two variables—gender and race. An item is considered biased if, for example, two respondents from different groups have different probabilities of answering in the same response category even though they have the same amount of the latent trait. Within the Rasch model, the test for DIF is specified by allowing the overall item difficulty to differ across demographic categories while controlling for the mean differences in the overall construct. The effect size for DIF with respect to gender was negligible for all items. For the race variable, comparisons were made for each group (Asian, Black, and Latino) to the White group, which is the subgroup that makes up the majority of people in STEM careers. Using the criteria provided by Paek (2009) and Paek and Wilson (2011), most items showed negligible DIF. However, Item 8 showed large DIF between students who identify as White over students who identify as Asian; Items 1, 2, 5, and 7 showed intermediate DIF between students who identify as White over students who identify as Black; and Item 9 showed intermediate DIF between students who identify as Black over students who identify as White (see Table 5).

## Discussion

This study contributes to the research on how high school students self-identify as researchers. This study’s aim was to develop a theory (i.e., a construct) of how high school students identify as researchers, and to design an instrument corresponding to that theory. To achieve this goal, a self-report instrument containing 12 items was developed and validated. Each item consisted of a stem and five ordered response options that corresponded to the waypoints defining the construct. A thorough investigation of the psychometric properties of the instrument was conducted. Overall, the findings from this study about the survey, the RISG, provide strong evidence for its reliability, validity, and fairness as a measurement tool.

### Reliability

We gathered multiple pieces of evidence of reliability to ensure consistency of the survey’s subscales and items, and person responses. The subscale correlations (disattenuated) ranged from 0.81 to 0.98. For items, the estimated reliability (coefficient alpha) of the researcher identity instrument at 0.91 was high and the EAP reliability was also high (also 0.91); and the reliability of person estimates within each of the strands was high ranging from 0.85 to 0.87. The evidence collected for this study indicates the RISG produces reliable results.

### Validity

A sound validity argument is informed by multiple strands of evidence. Therefore, we collected validity evidence based on the following sources, test content, response processes, internal structure, and relations to other variables, as delineated in the *Standards* (AERA et al., 2014) and reported on them in this study to constitute an empirical grounding for use of the instrument. This multi-evidence approach ensures the accuracy of our findings and provides a comprehensive bridging between the theory of researcher identity and the empirical results collected. Multiple pieces of empirical evidence support the quality of the instrument. The results from the meetings with experts indicate that the items map to the content (validity evidence based on test content). The focus group results of high school students provided validity evidence based on response processes. The survey results based on the partial credit model analysis, the response model parameter estimates, and the Wright map of items and persons, provided validity evidence for the internal structure of the instrument including empirical evidence in support of the waypoints (i.e., absent, curious, role exploration, comfortable with identity, and secure identity) of the construct. However, the analysis identified one item (Item 2) that was outside the typical range of acceptability. Finally, there was a positive correlation between the RISG and SQM-II (Glynn et al., 2011), which provided validity evidence for the survey’s relation to another variable. The investigations mentioned here provide strong evidence for the valid use of the instrument and interpretation and its results.

### Fairness

To investigate fairness, we used the survey data collected from high school students to conduct a DIF analysis. Within the Rasch model, the test for DIF is specified by allowing the overall item difficulty to differ across demographic categories while controlling for the mean differences in the overall construct. We investigated DIF for the gender and race variables. The effect size for DIF regarding gender was negligible for all items, and most items showed negligible DIF for race too except for Item 8. That item showed greater than expected DIF between students who identify as White over students who identify as Black. This is a concern and warrants monitoring.

### Broader aspects of the study

Our study is different from previous studies in several significant ways. First, we chose to investigate “researcher identity” because existing instruments were designed to measure “science,” “STEM,” and/or other identities and not researcher identity. Creating this survey filled a clear need for programs such as the authentic *research* experience. Second, by using a less loaded term than “STEM,” “science,” or similar, under represented populations, especially girls, may be more engaged although this needs further study. In addition, we primarily used Rasch modeling (Rasch, 1960/1980) to investigate the construct instead of a factor analysis approach, which has been the case in other studies (Glynn et al., 2011; Godwin et al., 2013; Dou et al., 2019; Cribbs et al., 2015; and Verdin et al., 2018). We took this approach because using IRT allowed us to focus on the measurement and evaluation of fit for the individual item (as well as the instrument as a whole). As mentioned, IRT models acknowledge the ordinal nature of the item response and allows the modeling of the step parameters instead of assuming that the raw scores are a continuous variable (as is the case with factor analysis).

With the call for an increase in authentic research experiences, programs such as the San Francisco Health Investigators have been growing in popularity, but these kinds of programs need valid, reliable, and fair tools for assessment. It is our aim that the instrument, the RISG, will be used in authentic research experience programs or research programs with similar content. Given that these types of programs are designed to attract and promote students’ interest in STEM, we see this research as contributing to the larger effort of widening the pathway into STEM careers.

## Conclusion

Central to this study was answering the question about the nature of the progression for self-identification as a researcher. Findings from this study about the survey, the RISG, provide strong evidence for its validity, reliability, and fairness as a measurement tool for assessing how high school students self-identify as researchers.

The advantages of the RISG are that the survey (a) was systematically developed to measure researcher identity, which makes it more directly applicable for use in research-oriented types of programs, (b) provides clear and interpretable results because of the underlying Guttman structure, and (c) can be easily interpreted without the use of statistical analysis because of the unique relationship the Rasch model has with raw scores.

Beyond the findings, this study demonstrates that the construct modeling approach, with an emphasis on the construct—and the construct map, is an effective approach to investigating the technical qualities of an instrument designed to measure an affective trait such as researcher identity. In particular, there is a direct relationship between the construct map and the item format (as shown in Fig. 4).

### Limitations and Recommendations for Future Studies

The study has limitations that could be addressed through further study. First, because of the innovative nature of the construct, further research should be conducted on the applicability and use of the companion instrument. Second, because role identities can be multifaceted and intersectional, research could be conducted to understand the prioritization and intermingling of those identities and how they influence decision-making on choosing fields of study at the undergraduate level, persistence to degree, and career choice. Third, this tool could be investigated for use by science teachers, counselors, academic advisors, and others. Additional research could include the development and validation of similar instruments for younger and older students. Finally, although Rasch analysis has the unique property of producing sample free estimations (Bond & Fox, 2015), the findings’ generalizability is limited because of our non-random sample.

## Supplementary Material

Researcher Identity Survey Morell et al 2024

## Figures and Tables

**Table 1:** Demographics of Research Participants

Variable			
Gender		Grade	
N	822	N	822
% Male	47	% in Grade 9	1
% Female	52	% in Grade 10	52
% Other	1	% in Grade 11	29
		% in Grade 12	19
Race		College Plan	
N	819	N	823
% Asian	40	% Undecided	9
% Black	4	% Plan to work	2
% Latinx	28	% 2-Year College	8
% White	20	% 4-Year College	80
% Other	8		

**Table 2:** Subscale correlations (disattenuated), and reliabilities*

Subscale				
Subscale (strands)	Self	Community	Agency	Fit & Aspiration
Self	*0.85*			
Community	0.95	*0.87*		
Agency	0.98	0.98	*0.87*	
Fit & Aspiration	0.81	0.89	0.80	*0.87*

Reliabilities are shown on the diagonal. The expected a posteriori (EAP) reliability = 0.91 and Coefficient alpha = 0.91

**Table 3:** Comparison between the One-dimensional & Multidimensional models

Model	AIC	BIC	Deviance	# of parameters
One-dimensional	24,383	24,614	24,285	49
Multidimensional	24,093	24,366	23,977	58

**Table 4:** Response Model Parameter Estimates for the Researcher Identity Items

Item	Difficulty Estimate^a^	Weighted Fit Mean Square^b^	*T* ^c^
1	1.361 (0.044)	0.89	−2.5
2	−0.120 (0.041)	1.39	7.6
3	1.170 (0.047)	0.84	−3.7
4	1.835 (0.047)	1.1	1.8
5	0.278 (0.039)	0.94	−1.3
6	0.419 (0.040)	1.27	5.4
7	0.505 (0.039)	0.91	−1.9
8	−0.064 (0.044)	1.23	4.5
9	0.947 (0.045)	1.00	−0.1
10	0.752 (0.040)	0.87	−3.0
11	0.254 (0.042)	0.87	−2.8
12	1.060 (0.045)	0.82	−3.8

a The difficulty estimate indicates the ability level required to have a probability of 0.50 of endorsing this item. The more difficult the item, the further the item characteristic curve is to the right. Respondents tend to endorse or agree with the items whose difficulty parameter is below their estimated location and not endorse the items whose difficulty estimate is above their level. The standard error of the estimate for each item is presented in parenthesis beside each item’s difficulty estimate

b The ideal weighted mean-square is 1.0 with a reasonable bound range of 0.75 to 1.33 (Adams & Khoo, 1996). When the observed item characteristic curve (ICC) is steeper than the expected ICC, the weighted mean-square is less than one, implying that the responses on the item are more predictable than expected by the model. Conversely, when the observed ICC is flatter than the expected ICC, the weighted mean-square is greater than one, indicating that the responses on the item are too unpredictable (less predictable than the model expects)

c A t-statistic outside the range of −1.96 to 1.96 indicates misfit at the 0.05 level. However, note that the t-statistic is sensitive to the sample size, and with a large sample size, even ignorable misfits will be flagged as significant (Wu & Adams, 2013), hence they should be interpreted in conjunction with effect sizes

**Table 5:** Differential Item Functioning (DIF) for the Race Variable

Item	Difficulty for White	Difficulty for Asian	Difficulty for Black	Difficulty for Latino	Asian vs. White^a^	Black vs. White^b^	Latino vs. White
Item 1	1.371 (0.088)	1.326 (0.090)	1.899 (0.088)	1.450 (0.088)	−0.045	0.528	0.079
Item 2	−0.432 (0.084)	0.021 (0.084)	0.138 (0.084)	−0.031 (0.084)	0.453	0.570	0.401
Item 3	1.184 (0.096)	1.046 (0.098)	1.211 (0.096)	1.416 (0.096)	−0.138	0.027	0.232
Item 4	1.794 (0.096)	1.843 (0.096)	2.097 (0.096)	1.890 (0.096)	0.049	0.303	0.096
Item 5	0.341 (0.080)	−0.016 (0.082)	0.825 (0.080)	0.589 (0.080)	−0.357	0.484	0.248
Item 6	0.265 (0.082)	0.492 (0.080)	0.116 (0.080)	0.521 (0.080)	0.227	−0.149	0.256
Item 7	0.493 (0.078)	0.305 (0.080)	0.967 (0.078)	0.782 (0.080)	−0.188	0.474	0.289
Item 8	−0.454 (0.092)	0.223 (0.090)	−0.118 (0.090)	−0.068 (0.090)	0.677	0.336	0.386
Item 9	0.802 (0.090)	1.081 (0.090)	0.311 (0.090)	1.030 (0.090)	0.279	−0.491	0.228
Item 10	0.956 (0.082)	0.615 (0.084)	0.848 (0.082)	0.877 (0.082)	−0.341	−0.108	−0.079
Item 11	0.273 (0.086)	0.082 (0.086)	0.232 (0.086)	0.446 (0.086)	−0.191	−0.041	0.173
Item 12	1.230 (0.092)	0.920 (0.092)	1.319 (0.092)	1.179 (0.092)	−0.310	0.089	−0.051

a Item 8 showed DIF between students who identify as White over students who identify as Asian

b Items 1, 2, 5, and 7 showed DIF between students who identify as White over students who identify as Black. Item 9 showed DIF between students who identify as Black over students who identify as White

## Data Availability

Data requests should be directed to the first author.
