# Supplementary material for: Developing and Gathering Validity Evidence for an Instrument to Measure How High School Students Identify as Researchers
Source: Res Sci Educ. Author manuscript; Available in PMC 2025 Jul 28. (PMC12303120; doi:10.1007/s11165-024-10194-1)
Supplement: Researcher Identity Survey Morell et al 2024 [file NIHMS2029499-supplement-Researcher_Identity_Survey_Morell_et_al_2024.pdf]

Supplementary Material

Researcher Identity Survey (RIS-G)

The survey measures the construct – Self-Identification as a Researcher. The table below defines qualitatively different levels of the degree to which a person self-identifies as a researcher.

**Title of Level**	**Description**
Secure Identity or Integration of Identity	Respondent identifies as researcher and integrates this into their larger self
Comfortable with Identity	Respondent begins to feel comfortable with their identity as a researcher
Role Exploration	Respondent explores the different aspects of research
Curious Identity	Respondent is a newcomer to the concept of research
Absent	Respondent is unaware of what research entails and has not considered their role in research

Developmental Progression of Researcher Identity for high school students

In addition, the Researcher Identity construct is composed of four strands. Those strands are defined as follows:

- Self – A person's current idea of self-identity as a researcher. The focus here is on how the student feels about the self at the present moment, Questions 1-3.
- Community – A person's sense of belonging to a research community, Questions 4-6.
- Agency - The degree to which a person feels empowered to impact change through research, Questions 7-9.
- Fit & Aspiration – A person's interest in research as a career path and belief in research as a great fit to their personal interests (future self), Questions 10-12.

This instrument is designed so that the scale is cumulative within each item block. This means that for each question-block, response options build from least (option a) through most identity as a researcher (option e). So, more "e" responses circled means that the respondent is higher on the Researcher Identity scale, and conversely, more "a" responses circled indicates that the respondent is lower on the scale. This instrument is valid for use with high school students.

Researcher Identity Survey (RIS-G)

The aim of this survey is to gain a better understanding of your perspective. There are no right or wrong answers. The survey should take about 10 minutes to complete. Please circle <u>one answer for each question</u>. Use the "researcher" definition below to answer each statement.

DEFINITION: A "researcher" is defined as someone who conducts an organized and systematic investigation on a topic or question related to a scientific field.

1. Which statement about being a researcher best captures your opinion of yourself?
   a) I do not consider myself a researcher.
   b) I hesitate to call myself a researcher.
   c) I am beginning to consider myself a researcher.
   d) I consider myself a student researcher.
   e) I consider myself to be a professional researcher.

2. Which statement below best describes your skills to do research?
   a) I do not have the skills to do research.
   b) I'm interested in gaining research skills.
   c) I have the skills to conduct research with a lot of help from others.
   d) I have the skills to conduct research with a little help from others.
   e) I have the skills to conduct research on my own.

3. Which statement below best captures your identity as a researcher?
   a) Being a researcher is not a part of who I am.
   b) I am not sure if being a researcher is a part of my identity.
   c) Being a researcher might be a small part of my identity.
   d) Being a researcher is a part of my identity.
   e) Being a researcher is a big part of my identity.

4. Which statement best describes you?
   a) I don't consider myself a part of a research community.
   b) I am beginning to feel like a part of a research community.
   c) I am a small part of a research community.
   d) I am a part of a research community.
   e) I am an important part of a research community.

5. Which statement best describes your interest in research?
   a) I do not have an interest to do research that helps my community.
   b) I am slightly interested in doing research that helps my community.
   c) I might be interested in doing research that helps my community.
   d) I would be interested in doing research that helps my community.
   e) I am definitely interested in doing research that helps my community.

6. Which statement best describes your level of comfort in communicating with researchers?
   a) I am uncomfortable speaking to experienced researchers right now.
   b) I hesitate to speak to researchers that have more experience than me.
   c) I am learning how to communicate with researchers that have more experience than me.
   d) I am comfortable talking to researchers that have more experience than me.
   e) I can speak with authority to researchers that have more experience than me.

7. Which statement best describes your interest in contributing to society?
   a) I do not have the desire to contribute to society through research.
   b) I have an interest in contributing to society through research.
   c) I have a desire to make some contribution to society through research.
   d) I have a desire to make a meaningful contribution to society through research.
   e) I have a strong desire to make a meaningful contribution to society through research.

8. Which statement best describes your level of skill?
   a) I have no research skills.
   b) I can research issues with a lot of help.
   c) I can research issues with some help.
   d) I can research issues with a little help from others.
   e) I can research issues independently.

9. Which statement best describes your level of *researcher voice*? We describe researcher voice as the extent to which you feel empowered to speak about your research.
   a) I do not want to have a *researcher voice*.
   b) I do not have a *researcher voice* now but I would like to develop one.
   c) I can use my voice to guide discussions.
   d) I am developing a strong *researcher voice*.
   e) I have a strong *researcher voice*.

10. Which statement best describes your future plans?
   a) I do not plan to pursue research in the future.
   b) I do not know if doing research is in my future.
   c) I am not sure if a research-related degree is right for me.
   d) I might get a research-related degree in college.
   e) I plan to get a research-related degree in college.

11. Which statement best describes your interest in research?
   a) I think research is boring.
   b) I think research is a little interesting.
   c) I think research is interesting.
   d) I think research is very interesting.
   e) I think research is an engaging field of study.

12. Which statement best describes your interest in a research career?
   a) A career in research would not be a good fit for me.
   b) I am not sure if I am interested in research as a career.
   c) I might have an interest in research as a career.
   d) A career in research might be a good fit for me.
   e) A career in research would be a great fit for me.

Scoring the RIS-G

Step 1.
Assign a number to each response option for each item using this system:
A = 0
B = 1
C = 2
D = 3
E = 4

Step 2.
Sum the 12 numbers together (you should have a number for each survey item) to get the *summed score*.

Step 3.
Locate the student's *summed score* within the specified ranges in the "Total Score" column. The level location indicates where the student is along the developmental trajectory. For example, if a student's *summed score* is 12, they would be at the "Role Exploration" level of the progression.

**RIS-G<br>Score Interpretation Guide for High School Students**		
**Total Score**	**Level**	**Description**
0-3	Absent	Respondent is unaware of what research entails and has not considered their role in research
4-8	Curious Identity	Respondent is a newcomer to the concept of research
9-21	Role Exploration	Respondent explores the different aspects of research
22-35	Comfortable with Identity	Respondent begins to feel comfortable with their identity as a researcher
36-48	Secure Identity	Respondent identifies as researcher and integrates this into their larger self

Note: The Scoring Interpretation Guide is based on the student answering all 12 items.
